# Emergency Medical Access Control System Based on Public Blockchain

**DOI:** 10.1007/s10916-024-02102-x

**Published:** 2024-09-19

**Authors:** Taisei Takahashi, Yan Zhihao, Kazumasa Omote

**Affiliations:** 1 Faculty of Engineering, Information and Systems, University of Tsukuba, Ibaraki, Japan; 2 Graduate School of Science and Technology, University of Tsukuba, Ibaraki, Japan

**Keywords:** Blockchain, Smart contract, Medical information sharing, Emergency response

## Abstract

IT has made significant progress in various fields over the past few years, with many industries transitioning from paper-based to electronic media. However, sharing electronic medical records remains a long-term challenge, particularly when patients are in emergency situations, making it difficult to access and control their medical information. Previous studies have proposed permissioned blockchains with limited participants or mechanisms that allow emergency medical information sharing to pre-designated participants. However, permissioned blockchains require prior participation by medical institutions, and limiting sharing entities restricts the number of potential partners. This means that sharing medical information with local emergency doctors becomes impossible if a patient is unconscious and far away from home, such as when traveling abroad. To tackle this challenge, we propose an emergency access control system for a global electronic medical information system that can be shared using a public blockchain, allowing anyone to participate. Our proposed system assumes that the patient wears a pendant with tamper-proof and biometric authentication capabilities. In the event of unconsciousness, emergency doctors can perform biometrics on behalf of the patient, allowing the family doctor to share health records with the emergency doctor through a secure channel that uses the Diffie-Hellman (DH) key exchange protocol. The pendant’s biometric authentication function prevents unauthorized use if it is stolen, and we have tested the blockchain’s fee for using the public blockchain, demonstrating that the proposed system is practical.

## Introduction

Blockchain, initially proposed by Satoshi Nakamoto [22], has expanded beyond finance into various industries like healthcare. The importance of medical information in the blockchain sector is growing, with an increasing number of publications [23]. Blockchain’s application in healthcare includes storing Electronic Health Records (EHRs) and enabling emergency access control.

For the blockchain-based emergency sharing system, the previous studies [21, 25] allow patients to specify in advance where their medical information will be shared. In an emergency, the selected entity can access the patient’s medical information. In summary, prior research has limited the entities that can access medical information in advance. Therefore, if an emergency occurs far from where the patient lives, the local emergency doctor cannot access the information and cannot help with urgent care. Limiting access rights is impractical unless the authorized entity is near the patient.

For example, consider the case of a British person with a pre-existing medical condition who loses consciousness while traveling in Vietnam and requires emergency medical care. In the previous study, it was necessary to specify in advance where the EHR would be shared in an emergency. Therefore, when a patient loses consciousness relatively close to home, the emergency doctor near the patient’s home can handle the situation using the method from the previous study. However, what about when the patient is abroad? The emergency doctor, who is a stranger to the patient and has not been designated in advance, will respond, making the previous study’s method ineffective. Our method does not require pre-specification. Thus, even if a patient loses consciousness abroad, the EHR can be sent to an unfamiliar emergency doctor.

This paper presents a new emergency medical access control system that is based on a public blockchain. It assumes that patients have a pendant with tamper-proof and biometric authentication capabilities, which would enable EHR sharing with emergency doctors anywhere in the world, even in remote locations. Our method involves encrypting patient EHRs by their family doctors and storing them in the InterPlanetary File System (IPFS). A smart contract is then used for emergency access control. Patients wear a pendant with tamper-proof and biometric authentication capabilities to enable emergency EHR retrieval by foreign doctors. This involves activating the smart contract, biometric authentication, secure key exchange, and EHR decryption. Our research integrates patient identification using a pendant with tamper-proof and biometric authentication capabilities and secure channel construction between medical institutions via blockchain-based smart contracts.

This paper will examine the emergency access control from the patient’s family doctor to the emergency doctor in the following ways:

1. The patient has a family doctor. The family doctor encrypts the patient’s EHR and stores it on the distributed file system, IPFS. Also, the family doctor deploys a smart contract for emergency access control and inputs his DH public key on the contract.
2. Patients wear a pendant with tamper-proof and biometric authentication capabilities. Suppose the worst-case scenario occurs such that the patient travels abroad and loses consciousness.
3. The emergency doctor in a foreign country wants to obtain the patient’s EHR from the family doctor. The emergency doctor uses biometrics and the device on behalf of the unconscious patient.
4. The emergency doctor uses the value output from the device to activate the smart contract and inputs his DH public key on the contract. After that, the emergency doctor obtains the EHR decryption key from the emergency doctor via a secure channel using DH shared secret. Finally, the emergency doctor can decrypt the ciphertext and obtain the EHR in plain text.

Our research involves a novelty technique of patient identification using tamper-proof devices with biometric authentication capabilities and constructing a secure channel between medical institutions through key sharing using public blockchain-based smart contracts.

## Related Work

Li  et al. [20] proposed a prototype data storage system on Ethereum blockchain to secure medical information from loss, theft, and tampering. Chen et al. [13] suggested a cloud-based blockchain system due to limitations in blockchain data storage. Fan et al. [15] introduced MedBlock, enhancing access and sharing of patient data via blockchain. MedRec [12] and MedChain [14] utilize blockchain and smart contracts for interoperability of medical records.

Ahmed et al. [25] introduced an Emergency Access Control Management System (EACMS) on permissioned based blockchain Hyperledger Composer, using smart contracts to manage PHR access during emergencies. Madine et al. [21] proposed a blockchain system for sharing emergency medical information, storing data in IPFS, and allowing partial or full transfer of access rights through proxy re-encryption.

## Preliminaries

### Electronic Health Records (EHR)

An *Electronic Medical Record (EMR)* is an electronic record of a patient’s medical care and is a digital version of paper documents and records in doctors and clinics. EMRs store data locally at the healthcare facility, whereas *EHR* aim to share health information among healthcare providers [17]. EHRs are more advanced regarding data sharing among healthcare providers, whereas EMRs include a partial patient history.

Note that, in this paper, the family doctor shares the patient’s medical data with the emergency doctor using blockchain. As it involves information sharing among medical institutions, the patient’s medical information is described using EHR notation.

### Blockchain

Blockchains are categorized as Public (Permissionless) or Permissioned based on transaction transparency and administrator presence.

Bitcoin [22] epitomizes public blockchains, allowing participation without permission and lacking an administrator. On the other hand, permissioned blockchains, come in two forms: private and consortium. Private blockchains have single administrators and limited user access, while consortium blockchains involve multiple administrators.

Garay  et al. [16] analyzed the backbone protocol of the Proof of Work-type blockchain. In [16], the analysis was based on the honest majority assumption that most users are honest. They defined *typical execution* where the results of honest and malicious miners do not deviate significantly from the central expectation. With sufficiently long runs, the probability of non-typical execution occurring can be considered negligibly small. Under the honest majority assumption and from typical execution, *k* can be regarded as a security parameter, and the larger *k* is taken, the more we can assume that the honest miners are in agreement on the same chain. In this paper, we adopt the honest majority assumption in the blockchain network and assume that the protocol is executed on a single chain where the consensus is ensured among the honest majority.

### Smart Contract

Smart contracts are a feature implemented in Ethereum, which has the next highest market value after Bitcoin and are automated programs that utilize blockchain technology to execute when conditions are met automatically. Stored on a reliable and tamper-proof blockchain, it increases the transparency of transactions and agreements and enhances trust without needing an intermediary.

Smart contracts are also part of decentralized applications and have a wide range of applications, including cryptographic assets, real estate transactions, and supply chain management. Smart contracts are typically used to automate contract execution so that all participants can see the results instantly without the involvement of intermediaries or loss of time.

Gas price refers to the cost of computational resources required to execute transactions and smart contracts on the Ethereum network. These costs are paid in the form of gas fees, which are determined by the amount of gas used to execute a specific process multiplied by the cost per unit of gas. Irrespective of the success or failure of the transaction, the gas fee must be paid in ETH, the native currency of Ethereum. The gas price is generally quoted in gwei, which is one of the units of ETH. One gwei is equivalent to one billionth of an ETH. For instance, a gas bill of 0.00000001 ETH can be represented as 10.0 gwei.

#### Testnet

*Testnet* is a blockchain environment that is not intended for production purposes. It is used to identify and fix bugs, code vulnerabilities, and other network issues that may impact the functionality of a blockchain project. To test smart contract functionality before deploying it on the production environment *Mainnet*, developers use testnets such as Rinkeby, Goerli, Sapolia, Polyscan, and Kiln. It is important to note that cryptocurrency on Mainnet has real value on the blockchain network and can be used to pay for transactions, but coins on testnet cannot be used on Mainnet. Transactions on Mainnet require real cryptocurrency, while transactions on testnet are free.

### InterPlanetary File System (IPFS)

The InterPlanetary File System (IPFS) [5] is a hypermedia protocol created by Juan Benet in 2015. It uses P2P networks developed by Protocol Labs. Unlike HTTP, the primary protocol used on the Internet, which is “location-oriented,” IPFS allows users to access content using a content ID (CID), which is the hash value of the content. This means that instead of specifying a URL of the web server providing the content, users can view the desired content by using its unique content ID.

### Tamper-Proof Hardware with Biometric Authentication Capabilities

Tamper-proof hardware refers to hardware devices and components that are highly resistant to external attack and physical manipulation, and are used to protect sensitive information and security-related tasks. Examples of tamper-proof hardware include Intel’s Software Guard Extensions (SGX) [4], as well as SIM cards and IC cards such as credit cards, which are widely used in the real world. Tamper-proof technology is also widely used in smartphones. It is implemented in the iPhone as “Secure Enclave [8]” and in Android devices as “Trusty TEE [10]”.

In general, tamper-proof hardware has the *de facto* standard “attested execution” functionality [24]. Algorithm 1 illustrates the “attested execution” function, where a digital signature key pair is used to ensure the integrity of executed programs within the hardware.

**Algorithm 1:**
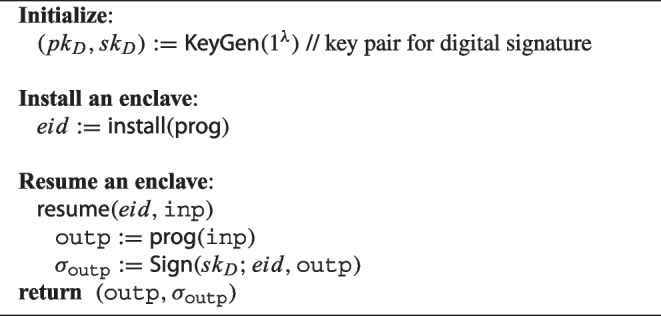
AESP’s brief algorithm.

First, the hardware manufacturer generates a digital signature key pair $(pk_D,sk_D)$. The output of any program executed in the hardware is signed with $sk_D$. Next, the manufacturer installs an arbitrary program $\textsf{prog}$ to run in the hardware. Finally, the program is executed. The program receives $\texttt{inp}$ and outputs $\texttt{outp}$. The $\texttt{outp}$ is signed with the digital signature key $sk_D$, and the signature $\sigma _\texttt{outp}$ is generated. The signature $\sigma _\textsf{prog}$ allows us to verify that the output $\texttt{outp}$ was successfully computed and generated from the tamper-proof device and $\texttt{outp}$ has not been tampered with.

We assume that our design of the pendant includes a SIM card, which is a portable hardware component, and that it communicates with the emergency doctor using Bluetooth, a short-range wireless communication standard for digital devices. For biometric authentication, we assume the use of methods such as fingerprint recognition, which is already widely used in smartphones and other devices. This method can be implemented on lightweight hardware with limited computing power [28] and can be used even if the patient is unconscious.

### Diffie-Hellman (DH) Key Exchange protocol

DH public-key cryptography is a cryptographic protocol in which two parties each output a public key, from which a shared secret key is created only between them. Consider the case where Alice and Bob output their public keys to each other to create the shared secret key.

1. Alice input $1^\lambda$ to a probabilistic polynomial time algorithm $\mathcal {G}$ and then obtains a cyclic group $\mathbb {G}$ of order *q*.
  1 $$\begin{aligned} (\mathbb {G}, g, q) \leftarrow \mathcal {G}(1^\lambda ) \end{aligned}$$
  where $\lambda$ is a security parameter.
2. Alice chooses $x \in \mathbb {Z}_q$ uniformly random and computes her public key as follows:
  2 $$\begin{aligned} pk_A = g^{x}. \end{aligned}$$
  Then, Alice sends $\left( (\mathbb {G}, g, q), pk_A \right)$ to Bob.
3. Bob chooses $y \in \mathbb {Z}_q$ uniformly random and computes his public key as follows:
  3 $$\begin{aligned} pk_B = g^{y}. \end{aligned}$$
4. Alice and Bob calculate the shared secret key, respectively.
  4 $$\begin{aligned} {\begin{matrix} sk_{AB} = (g^x)^y = (g^y)^x = g^{xy}. \end{matrix}} \end{aligned}$$

## Proposed Architecture

**Fig. 1:**
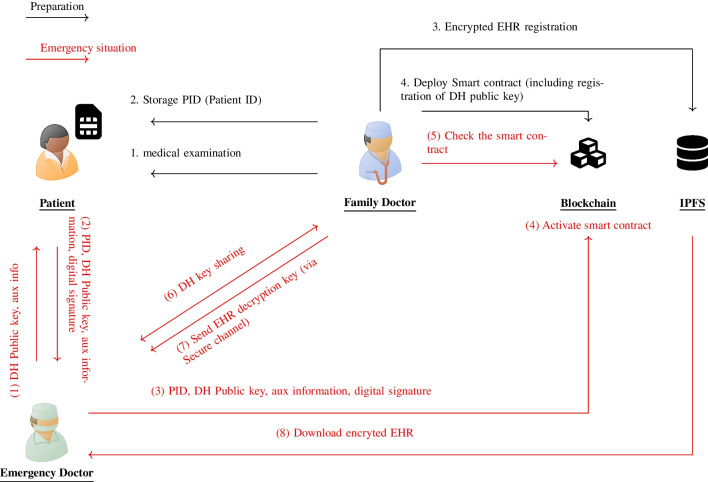
Overall Diagram

Figure 1 overviews our proposed emergency access control system. The system involves three characters: a patient, a family doctor, and an emergency doctor. We assume that the patient has a chronic disease and regularly visits their family doctor. They also have a pendant with tamper-proof and biometric authentication capabilities, which is capable of attested execution. The output from the pendant is digitally signed, making it possible to verify that it indeed comes from the patient’s pendant. Note that we assume that the patient pays all the transaction costs since the patient is the beneficiary of our proposal.

### Preparation

After examining the patient, the family doctor encrypts their medical data using a symmetric key cipher and stores it in the IPFS. To prepare for emergency response, the doctor deploys a smart contract on the blockchain. This smart contract stores the doctor’s DH (Diffie-Hellman) public key. Note that the original encrypted data stored in the IPFS is kept by the family doctor. Therefore, the original EHR data will not be lost if the family doctor loses his private key. The reason not to store all data in the IPFS is to allow only selected data to be shared. Some patients may be hesitant to disclose all information to an unfamiliar emergency doctor, so data can only be shared to the extent agreed upon in advance between the patient and the family doctor.

Additionally, the doctor deploys the public key, which corresponds to the private key for the digital signature on the pendant, to the smart contract. The smart contract can only be executed if it can verify the digital signature of the input and confirm that it was sent from the pendant.

### Emergency Situation

Consider a situation in which a British traveler suffers a sudden loss of consciousness in Vietnam and requires immediate medical attention. In such emergencies, local doctors need to access the patient’s medical data quickly to provide appropriate treatment. To facilitate this, the emergency doctor inputs their public key and contact details, such as an email address, into the patient’s pendant. The pendant also outputs a pre-installed unique Patient ID (PID), which is used by the doctor to identify the patient and is digitally signed.

To activate the smart contract, the pendant must produce a digitally signed output and pass the patient’s biometric authentication. Since passing biometrics indicates that an unusual event has occurred, requiring the EHR to be sent, the family doctor needs to respond to the EHR sharing.

The outputs and the digital signature activate a smart contract, enabling secure communication between the patient’s family and emergency doctors. Through a Diffie-Hellman key exchange, doctors establish a secure channel for sharing decryption keys. With this key, the emergency doctor can retrieve and decrypt the patient’s medical records from IPFS, enabling prompt and informed medical intervention.

Note that the family doctor sends the decryption key to the emergency doctor because the large size of the EHR data may make direct transmission difficult.

### Key Preparation

This section describes the keys used in this paper. See the notation in Table 1.

- $sk_D$: Secret keys for digital signatures built into the pendant. The program output is digitally signed by this key in the hardware.
- $pk_D$: The public key corresponds to the private key $sk_D$. This key is used in the smart contract to verify that the output is from the pendant.
- $pk_{F}, pk_{E}$: The public keys for a family doctor and an emergency doctor of DH key sharing protocol, respectively.
- $sk_{S}$: The shared secret key obtained by the family doctor and the emergency doctor generated from $pk_{F}$ and $pk_{E}$. This key is used to establish a secure channel between the two parties to enable secure information exchange.
- $sk_{F}$: The symmetric encryption key generated by the family doctor to encrypt and upload the patient’s medical records on IPFS.

**Table 1:** The list of keys

	Description	Location, Owner
*m*	Patient’s EHR	Generated by a family doctor and located on IPFS
$\textsf{auxinfo}$	contact information (*e.g.,* e-mail address)	Emergency doctor sends this information to the pendant
$sk_D$	Secret key for digital signature	Pendant (Tamper-proof)
$pk_D$	Public key for digital signature	Blockchain, Smart contract
$pk_{F}, pk_{E}$	Public keys for DH key sharing protocol	Family doctor and Emergency doctor, respectively
$sk_{S}$	DH shared secret key	Family doctor and Emergency doctor
$sk_{F}$	Symmetric encryption key for encrypting Patient’s EHR	Family doctor

#### Detailed Flow for Preparation

This section explains how to prepare for emergency access control of medical information. When patients visit their family doctor, the doctor examines them and saves their medical records. The doctor then encrypts the patient’s medical data and saves it in IPFS.

1. Medical checkup: The patient goes to the family doctor for a medical examination, and the doctor records the examination results *m*.
2. Registration of PID: The family doctor assigns a PID to each patient by generating a random number and stores the PID in the patient’s pendant.
3. Encryption and registration of EHR: The family doctor issues a symmetric encryption key $sk_{F}$ for each patient, then encrypts the patient’s EHR *m*. Then, the doctor registers the encrypted EHR $c_m$ in IPFS.
  5 $$\begin{aligned} c_m = \textsf{Enc}(sk_F; m). \end{aligned}$$

#### Detailed Flow for Emergency Situation

1. Examination (in case of emergency): When a patient is in an emergency situation and is unable to operate a medical device themselves, an emergency doctor takes charge and operates the device on their behalf. To do so, the emergency doctor inputs and activates the pendant, $\texttt{inp}$ contains their DH public key and other necessary information. This ensures that the device can be used safely and effectively to treat the patient in need.
  6 $$\begin{aligned} \texttt{inp}= \{ pk_{E}, \mathsf {aux \, info} \} \end{aligned}$$
  The auxiliary information $\textsf{auxinfo}$ contains the contact information (*e.g.,* e-mail address) to build a secure channel later between family and emergency doctor.
2. Activate smart contract with pendant’s output: The emergency doctor activates the smart contract with the $\texttt{outp}$ and $\sigma _{\texttt{outp}}$ from the pendant, allowing verification that the $\texttt{outp}$ is from the patient’s pendant. The $\texttt{outp}$ contains the PID, the emergency doctor’s DH public key, and signatures of auxiliary information.
  7 $$\begin{aligned} \begin{aligned} \texttt{outp}&=\textsf{prog}(\texttt{inp}) \\ \sigma _\texttt{outp}&=\textsf{Sign}(sk_D; \texttt{outp}) \end{aligned} \end{aligned}$$
  where
  8 $$\begin{aligned} \texttt{outp}= \{ pk_{E}, \mathsf {aux \, info}, PID \}. \end{aligned}$$
3. Confirmation of smart contract activation: The family doctor checks the verification result of the $\sigma _\texttt{outp}$, and if the verification is successful, the doctor recognizes the executor as an emergency doctor.
4. DH key sharing: DH key sharing is performed between the family and the emergency doctor, and both parties obtain the shared secret key $sk_{S}$. Then, both parties established the secure channel using $sk_{S}$.
5. Send decryption key for EHR: The family doctor sends $sk_F$ to the emergency doctor via the secure channel as follows:
  9 $$\begin{aligned} c_{sk_{F}} =\textsf{Enc}(sk_S; sk_F). \end{aligned}$$
  Then, the emergency doctor decrypts $c_{sk_{F}}$ and obtains $sk_{F}$ as follows:
  10 $$\begin{aligned} sk_{F} = \textsf{Dec}(sk_S; c_{sk_{F}}). \end{aligned}$$
6. Downloads and decrypts EHR: The emergency doctor downloads the EHR from IPFS and decrypts it:
  11 $$\begin{aligned} m = \textsf{Dec}(sk_{F}; c_m). \end{aligned}$$

#### DH Public Key Listing

The algorithm for posting the doctor’s DH public key on the smart contract is described in Algorithm 2. The shared secret key is generated by the public key of the family doctor and the emergency doctor

**Algorithm 2:**
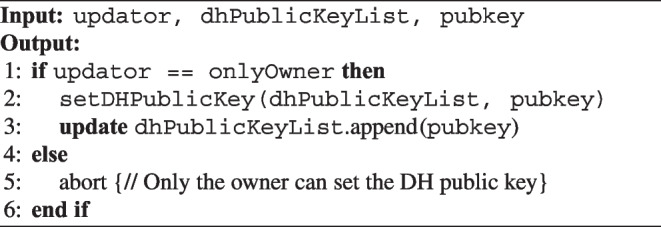
An algorithm for posting the doctor’s DH public key.

In this smart contract algorithm, the account that deploys the contract can set the DH public key, which is stored in dhPublicKeyList. The function setDHPublicKey is used to set and retrieve the DH public key, and the owner uses this function to set the DH public key. The onlyOwner modifier only sets the DH public key for the deployed account.

#### ECDSA Signature Verification

Algorithm 3 describes how to verify the digital signature output from the patient’s tamper-proof device on the smart contract. After successful verification, the emergency doctor’s DH public key is published to the smart contract.

**Algorithm 3:**
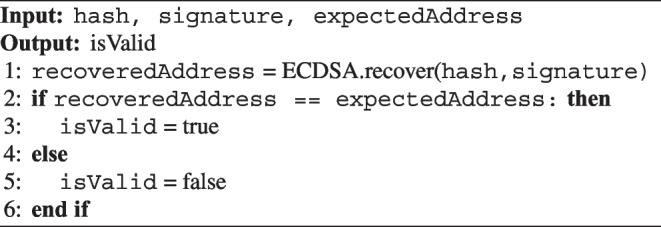
Verification of ECDSA signature.

First, the contract imports the ECDSA library. The ECDSA signature was verified using the ECDSA library from OpenZeppelin [6]. The emergency doctor obtains the hash and the signature and checks the function’s return value. If the public key matches the provided acquisition, true is returned as the signature is valid.

Algorithm 3 is the code in the smart contract, which uses ECDSA.recover to verify that the signature is from the pendant according to the Solidity specification. ECDSA normally uses public keys to verify signatures, but smart contracts used in Ethereum do not require public keys for signature verification. This is because ECDSA has a public key recovery function, allowing the public key to be recovered internally for signature verification. The wallet address is used to verify the validity of the public key. Therefore, it is necessary to provide the wallet address for signature verification instead of the public key.

## Performance Evaluation

Our proposed method utilizes a public blockchain, which incurs a Gas cost, that is, the transaction fee required for executing the smart contract. To measure the Gas cost incurred, we have deployed our implementation in the Ethereum testnet environment called Sepolia [1]. For smart contract development, we have used Solidity as the language and Metamask as the Ethereum wallet, which is available as a web browser extension.

We have implemented the smart contract using Infura [3], which provides node hosting services to connect to the Ethereum environment, and Remix [7], an integrated development environment to develop smart contracts.

Our experimental environment used was a Microsoft Surface laptop3 (processor: Intel(R) Core(TM) i5-1035G7 CPU @ 1.20GHz 1.50GHz, memory: 8.00 GB (7.60 GB available)).

### Experimental Methods

In this experiment, we have measured two locations where gas costs are incurred during implementation in the testnet. We have measured two points:

1. The cost of deploying the smart contract for registering DH public keys of emergency doctors.
2. The execution of the smart contract by the emergency doctor, including verifying the ECDSA signature.

### Experimental Results

**Table 2:** Gas costs for deployment and smart contract execution

	Gas fee
Smart contract deployment by the family doctor	3.48 Gwei
ECDSA authentication in the smart contract	2.50 Gwei

The experiment results are shown in Table 2. The gas cost for the family doctor to post the DH public key to the smart contract was 3.48 Gwei. The cost of ECDSA authentication by the emergency doctor was 2.50 Gwei. Gas prices in Ethereum vary according to the price of Ehther; as of March 2024 [11], 1 Gwei is roughly 0.034 USD. The DH public key deployment is 1.5 Gwei, roughly 0.05 USD. The ECDSA verification is 2.50 Gwei, which is roughly 0.16 USD. We found that the Gas cost of using a public blockchain was meager, low-cost, and practical.

## Discussion

### Reality of Tamper-Proof Hardware Assumption

If the pendant with tamper-proof and biometric authentication capabilities is not used, the smart contract should have a pre-defined list of emergency doctors worldwide, a public key for digital signatures, and a DH public key. This is necessary for authenticating emergency doctors on the smart contract.

However, this approach has its limitations. It requires the posting of all emergency doctors worldwide in the smart contract, which can be costly. Additionally, maintaining a list of all doctors or medical institutions is similar to using a permissioned blockchain and is practically impossible to achieve.

### Potential for Abusing the Pendant with Tamper-proof and Biometric Authentication Capabilities

The patient’s pendant is equipped with tamper-proof hardware to ensure its safety and security. However, in case of an emergency in which the patient loses consciousness, there is a risk of the pendant being stolen and misused by an adversary.

The pendant comes with a biometric authentication function to prevent unauthorized use. Even if the pendant is stolen, the feature ensures that the adversary cannot use it without authorization.

### Emergency Response Time

It is crucial to start curative treatment within one hour from the time of injury in trauma treatment. This first hour is known as the “golden hour” and is considered a critical period that can mean the difference between life and death [19].

Since Ethereum usually approves transactions in just 15 seconds, generating blocks and processing transactions within one hour is considered sufficient time.

### Restrict Access to IPFS

Our proposed method can be implemented if at least IPFS and e-mail are available. In some countries, access to the Internet is strongly restricted. No country seems to have explicitly stated any particular restriction on IPFS access. Even in China, where access restrictions have been tightened, it appears that IPFS has not been blocked and is still available for use [9]. However, it is impossible to use our proposed method in a limited number of countries, such as North Korea, where Internet access is blocked from the outset.

### Limitation

#### Unethical Doctors

Since the family and emergency doctors own the patient’s EHR, unethical doctors may sell or share this data without the patient’s consent and without traceability. The sale of data by unethical doctors is impossible to avoid as long as the doctor makes a diagnosis or views the EHR.

However, even in other countries, emergency doctors can be traced back to a family doctor. If the emergency doctor sells the EHR, the family doctor may be able to identify the emergency doctor who sold the data. Thus, the possibility of being traced acts as a deterrent to crime for the emergency doctor.

#### EHR Sharing Partner Verification

Since the pendant has a biometric authentication function, the EHR will not be shared even if it is stolen. However, if the patient is asleep or restrained, the biometric authentication may be bypassed without the patient’s consent, potentially leading to improper access to the EHR. A major challenge with our method is the inability to confirm that the person with whom the EHR is shared is indeed a healthcare professional. This is because our method allows sharing the EHR without pre-specifying the recipient; as long as the pendant passes the biometric authentication, the EHR can be shared with anyone.

A possible solution is to verify that the EHR is shared with a healthcare professional. One possible solution is to use Self-Sovereign Identity (SSI) to verify that the emergency doctor is indeed a healthcare professional. SSI is a technology that manages identities in a self-sovereign manner without relying on a third party [18]. In a previous study, SSI using the blockchain is used to verify the identity of a medical professional [26].

In our method, using SSI to reveal the identity of emergency doctors could make it possible to verify that the person with whom the EHR is shared is a medical professional.

## Conclusion

We have developed a new system for accessing emergency medical information using a global blockchain. Patients’ family doctors encrypt their medical records using IPFS, and patients themselves wear the pendants for identity verification. In the event of an emergency, doctors can activate smart contracts using the patient pendants to access the medical records needed for immediate treatment.

The advantage of our method is that both family doctors and emergency doctors can automate their work. The application can perform all of the family doctor’s tasks, from updating the IPFS and checking the activation of the smart contract to sending the data. Thus, even if the family doctor is asleep or has temporal issues, the EHRs can be shared with the emergency doctor automatically and rapidly. Additionally, simplifying emergency doctor tasks such as blockchain transactions through user-friendly smartphone apps would increase operational efficiency. In this paper, we have tested the system’s functionality on the Ethereum testnet. Going forward, it will be important to standardize the sharing of international medical data, for example through HL7 FHIR [2, 27].

Our proposed method has the advantage that it can be shared not only by one family doctor but also by several doctors, such as specialists. It is possible for one doctor to consolidate data from several doctors into one. Alternatively, it is possible to share data from several doctors via different routes by separating smart contracts for each doctor.

## Data Availability

No datasets were generated or analysed during the current study.

## References

[1] ETHEREUM SEPOLIA FAUCET. https://sepoliafaucet.com/

[2] FHIR v5.0.0. https://hl7.org/fhir/

[3] Infura. https://www.infura.io/

[4] Intel Software Guard Extensions. https://www.intel.com/content/www/us/en/developer/tools/software-guard-extensions/overview.html

[5] IPFS: An open system to manage data without a central server. https://ipfs.tech

[6] Openzeppelin-securely code, deploy and operate your smart contracts. https://www.openzeppelin.com/

[7] Remix-Ethereum IDE. https://remix.ethereum.org/

[8] Secure Enclave. https://support.apple.com/en-gb/guide/security/sec59b0b31ff/web

[9] South China Morning Post - Web3 tech helps banned books on piracy site library Genesis slip through the Great Firewall’s cracks, but for how long? https://www.scmp.com/tech/tech-trends/article/3172431/web3-tech-helps-banned-books-piracy-site-library-genesis-slip

[10] Trusty TEE. https://source.android.com/docs/security/features/trusty

[11] WalletInvestor. https://walletinvestor.com/converter/gas/ethereum/

[12] Azaria, A., Ekblaw, A., Vieira, T., and Lippman, A., MedRec: Using blockchain for medical data access and permission management. In: *2016 2nd International Conference on Open and Big Data (OBD)*, pp. 25–30, 2016. 10.1109/OBD.2016.11

[13] Chen, Y., Ding, S., Xu, Z., Zheng, H., and Yang, S., Blockchain-based medical records secure storage and medical service framework. *J. Med. Syst.* 43(1):1–9, 2018. 10.1007/s10916-018-1121-4 30467604 10.1007/s10916-018-1121-4

[14] Daraghmi, E. Y., Daraghmi, Y. A., and Yuan, S. M., MedChain: A design of blockchain-based system for medical records access and permissions management. *IEEE Access* 7:164595–164613, 2019. 10.1109/ACCESS.2019.2952942

[15] Fan, K., Wang, S., Ren, Y., Li, H., and Yang, Y., MedBlock: Efficient and secure medical data sharing via blockchain. *J. Med. Syst.* 42(8):136, 2018. 10.1007/s10916-018-0993-7 29931655 10.1007/s10916-018-0993-7

[16] Garay, J., Kiayias, A., and Leonardos, N., The bitcoin backbone protocol: Analysis and Applications. In: *Advances in Cryptology - EUROCRYPT 2015, Lecture Notes in Computer Science*, pp. 281–310. Springer, 2015. 10.1007/978-3-662-46803-6_10

[17] Heart, T., Ben-Assuli, O., and Shabtai, I., A review of PHR, EMR and EHR integration: A more personalized healthcare and public health policy. *Health Policy Technol.* 6(1):20–25, 2017. 10.1016/j.hlpt.2016.08.002

[18] Houtan, B., Hafid, A. S., and Makrakis, D., A Survey on blockchain-based self-sovereign patient identity in healthcare. *IEEE Access* 8:90478–90494, 2020. 10.1109/ACCESS.2020.2994090

[19] Kotwal, R. S., Howard, J. T., Orman, J. A., Tarpey, B. W., Bailey, J. A., Champion, H. R., Mabry, R. L., Holcomb, J. B., and Gross, K. R., The effect of a golden hour policy on the morbidity and mortality of combat casualties. *JAMA Surgery* 151(1):15–24, 2016. 10.1001/jamasurg.2015.3104 26422778 10.1001/jamasurg.2015.3104

[20] Li, H., Zhu, L., Shen, M., Gao, F., Tao, X., and Liu, S., Blockchain-based data preservation system for medical data. *J. Med. Syst.* 42(8):141, 2018. 10.1007/s10916-018-0997-3 29956058 10.1007/s10916-018-0997-3

[21] Madine, M. M., Salah, K., Jayaraman, R., Yaqoob, I., Al-Hammadi, Y., Ellahham, S., and Calyam, P., Fully decentralized multi-party consent management for secure sharing of patient health records. *IEEE Access* 8:225777–225791, 2020. 10.1109/ACCESS.2020.3045048

[22] Nakamoto, S., Bitcoin: A peer-to-peer electronic cash system. https://bitcoin.org/bitcoin.pdf

[23] Omote, K., Inoue, Y., Terada, Y., Shichijo, N., and Shirai, T., A Scientometrics Analysis of Cybersecurity Using e-CSTI. *IEEE Access* 12:40350–40367, 2024. 10.1109/ACCESS.2024.3375910

[24] Pass, R., Shi, E., and Tramèr, F., Formal abstractions for attested execution secure processors. In: *International conference on the theory and applications of cryptographic techniques – eurocrypt 2017, Lecture notes in computer science*, pp. 260–289. Springer International Publishing, 2017. 10.1007/978-3-319-56620-7_10

[25] Rajput, A. R., Li, Q., Taleby Ahvanooey, M., and Masood, I., EACMS: Emergency access control management system for personal health record based on blockchain. *IEEE Access* 7:84304–84317, 2019. 10.1109/ACCESS.2019.2917976

[26] Saha, S., Nova, S. N., and Iqbal, M. I., Healthcare professionals credential verification model using blockchain-based self-sovereign identity. In: *Proceedings of the fourth international conference on trends in computational and cognitive engineering*, pp. 381–392. Springer Nature, 2023. 10.1007/978-981-19-9483-8_32

[27] Saripalle, R., Runyan, C., and Russell, M., Using HL7 FHIR to achieve interoperability in patient health record. *J. Biomed. Inform.* 94:103188, 2019. 10.1016/j.jbi.2019.103188 10.1016/j.jbi.2019.103188 31063828

[28] Yang, W., Wang, S., Zheng, G., Yang, J., and Valli, C., A privacy-preserving lightweight biometric system for internet of things security. *IEEE Commun. Mag.* 57(3):84–89, 2019. 10.1109/MCOM.2019.1800378

